# Effect of ultra-fast high-intensity light-curing on the properties of a new bulk-fill restorative resin composite system: A Scoping Review

**DOI:** 10.4317/jced.61661

**Published:** 2024-07-01

**Authors:** Samille-Biasi Miranda, Maria-Luiza-Araújo-de Oliveira-Pinho Alves, Luiz-Antônio-Soares Falson, Caroline-de Farias-Charamba Leal, Ana-Karina-Maciel de Andrade, Rodrigo-Barros-Esteves Lins, Marcos-Antonio-Japiassú-Resende Montes

**Affiliations:** 1 MsC Student. Department of Dental Materials, Faculty of Dentistry, University of Pernambuco, Recife, Pernambuco, Brazil; 2 Undegraduation Student. Faculty of Dentistry, University of Pernambuco, Recife, Pernambuco, Brazil; 3 MsC Student. Department of Semiology and Clinical, Faculty of Dentistry, Federal University of Pelotas, Pelotas, Rio Grande do Sul, Brazil; 4 PhD Student. Faculty of Dentistry, University of Pernambuco, Recife, Pernambuco, Brazil; 5 Associate Professor. Department of Restorative Dentistry, Faculty of Dentistry, Federal University of Paraíba, João Pessoa, Paraíba, Brazil; 6 Adjunct Professor. School of Dentistry, Federal University of Alagoas, Maceió, Alagoas, Brazil; 7 Associate Professor. Department of Dental Materials. Faculty of Dentistry, University of Pernambuco, Recife, Pernambuco, Brazil

## Abstract

**Background:**

This scoping review aims to analyze the impact of rapid high-intensity light-curing on a new bulk-fill resin-based composites (RBCs) designed for this type of polymerization.

**Material and Methods:**

This scoping review was reported according to the Preferred Reporting Items for Systematic reviews and Meta-Analyses extension for Scoping Reviews (PRISMA-ScR) and Joanna Briggs Institute Manual of Evidence Synthesis. The methods were registered on the Open Science Framework (<osf.io/pq57t>). The literature search was conducted in PubMed/MEDLINE, Embase, Web of Science, Scopus and Cochrane Library databases. Eligibility was considered for in vitro and clinical studies evaluating the effects of ultra-fast high-intensity light-curing on a new system of bulk-fill RBCs.

**Results:**

Of 1.688 articles identified, 27 were included in the qualitative synthesis. All studies were conducted *in vitro*. A total of 2.432 specimens were evaluated. The studies have shown that shortness light-curing may result in similar properties (stress generated by polymerization shrinkage, marginal integrity, and bond strength to dental interface) for the new bulk-fill RBCs.

**Conclusions:**

Therefore, the new bulk-fill RBCs can be light-cured with a short exposure time and high intensity, providing a time-saving benefit in clinical practice, with similar results to standard light-curing in conventional composites, although, its use should be approached with caution in the flowable composite.

** Key words:**Resin composites, polymerization, dental materials, review.

## Introduction

Dentists commonly use resin composites in direct dental restorations because of their ability to replicate the optical characteristics of natural teeth (1). However, current protocols demand the use of a 2 mm incremental technique, causing prolonged clinical time and light irradiation in several increments, rendering this technique more sensitive and time consuming (2). Dental manufacturers constantly make changes to the formulation of restorative materials to improve properties, such as fluidity and translucency, try new combinations of photoinitiators and change the content and shape of the load, to ensure adequate polymerization and reduce errors during the clinical protocol (3-5).

In the last decade, a new category of composite resins known as bulk fill resins has emerged. These resins have lower polymerization stress compared to conventional resins, which allows them to be effectively light-cured in increments of up to 4 mm (6-8). Laboratory studies, such as those evaluating the stress generated by polymerization shrinkage (9), have highlighted the promising performance of bulk-fill resin-based composites (RBCs) compared to conventional resins, particularly in terms of marginal discoloration and adaptation (10,11), clinically similar results were observed regarding brightness, color stability, and translucency (12,13).

Photopolymerization has been a subject of study over the years, particularly aiming to reduce the photoactivation time of composite resins. One of the most recent proposed measures is the increase in irradiance of light curing units (LCUs), where a 3-second photoactivation becomes similar to activation times of 40 and 60 seconds (14). In response to this, restorative materials are being developed to meet these demands through a new polymerization approach that combines high irradiance with a short exposure time to light (14,15). Changes in the polymerization mechanism were carried out with the intention of shortening the exposure time, through the implementation of reversible addition-fragmentation chain transfer (RAFT) polymerization (16).

In this scenario, modified bulk-fill RBCs have emerged in the market, available in both sculpTable and flow consistencies. They have been enhanced with RAFT technology and improved characteristics of photoinitiation, translucency, and filler content, enabling ultra-fast photopolymerization for three seconds with a luminous emittance of 3050 mW/cm² (17). This innovative behavior is attributed to the addition of the β-allyl sulfone reagent, which facilitates polymerization in stages with shorter polymer chains and a more homogeneous polymerization (17). In some cases, the only alteration occurred in ensuring high translucency of particles in the pre-cure state, low filler content, and the addition of Ivocerin photoinitiator in their formulation, capable of enhancing light absorption and reducing polymerization time (18,19), proving more effective than Camphorquinone, commonly found in conventional composites (20).

The introduction of this new generation of bulk-fill RBCs has garnered attention from a clinical standpoint (21). While there are concerns regarding increased polymerization shrinkage stress, marginal integrity (22), and the potential for temperature rise due to high light intensity, which could lead to pulp and soft tissue damage (14), it is essential to analyze the behavior of these materials and their potential clinical benefits and hazards (3). This scoping review aims to comprehensively map the scientific literature regarding the impact of rapid high-intensity light-curing on the behavior of newly developed bulk-fill RBCs, specifically engineered for this advanced polymerization technique. By doing so, it addresses the pressing need for a deeper understanding of the clinical implications associated with these innovative materials. Moreover, the findings of this study can provide invaluable guidance to dental professionals in selecting the most suitable materials, fostering evidence-based clinical practice. Additionally, it offers insights for future research endeavors, promoting knowledge sharing and the ongoing improvement of restorative dentistry.

## Material and Methods

-Protocol and registration

This scoping review was conducted and reported according to the Preferred Reporting Items for Systematic Reviews and Meta-Analyses (Extension for Scoping Reviews) (23) and the Joanna Briggs Institute Manual of Evidence Synthesis (24). The protocol is registered in the Open Science Framework (<osf.io/pq57t>). As the new generation of Bulk Fill RBCs was recently introduced on the market, the scope review methodology was chosen, as it is suited for mapping more broadly and utilizing various types of evidence, addressing subjects that require deeper understanding, and thus identifying gaps for more specific future studies.

-Eligibility criteria 

The PCC strategy (population, concept and context) (23) was used (P: resin composites); (C: bulk- fill resin composites); and (C: rapid high-intensity light curing) which also based the research question: “What are the effects of rapid high-intensity light-curing on a new system of bulk fill RBCs?” Inclusion criteria were experimental *in vitro* and clinical studies (randomized and non-randomized clinical trials) that investigated the effect of ultra-fast light curing (1, 3 and 5s) on the behavior of new bulk-fill RBCs compared to standard light curing (10 and 20s) of conventional resin composites. Exclusion criteria were letters to the editor, literature reviews, clinical case reports and case series, manufacturer documents, conference abstracts and studies that have not evaluated the behavior of new bulk-fill RBCs with the rapid high-intensity light-curing protocol and studies published in languagens other than English.

-Search strategy

The electronic search was performed in September 2023 by two reviewers (SBM and MLAOPA) of the PubMed/Medline, Embase, Web of Science, Scopus and The Cochrane Library databases. A search strategy was performed in the PubMed/MEDLINE database and adapted for other databases, as show in  Table 1. The references of included articles were inspected to identify additional eligible studies. No search filters were used in this review.

-Study Selection

Articles were selected by two reviewers (SBM and MLAOPA) independently and blindly without filtering by year of publication or language. All identified articles were exported to Rayyan Management Software (Qatar Computing Research Institute, Doha, Qatar). After removing duplicates, titles and abstracts were read, and the eligibility criteria were applied. Potential articles were read in full before inclusion or exclusion. In cases of disagreement between the two reviewers, a consensus was reached through discussion with a third reviewer (RBEL). The level of agreement between the reviewers for the inclusion of studies was estimated based on the Kappa Score (25).

-Data collection

Two reviewers (SBM and MLAOPA) collected and interpreted the data using a standardized Excel spreadsheet (Microsoft Corporation, Redmond, Washington, USA). A consensus meeting with a third reviewer (RBEL) confirmed the extracted data. The variables collected from the studies were author’s name, year of publication, restorative composites evaluated, sample number, unit of study, intervention, and main results about the new bulk-fill RBCs; in addition to data referring to the light curing unit (LCU), LCU evaluation method, radiant exposure, emission spectrum and irradiation time.

-Data analysis

A qualitative and detailed synthesis of the data extracted from studies that met the eligibility criteria was performed. Tables of individualized results were formulated for the included studies and data related to the most relevant characteristics of these studies. Since composite resins polymerized with high intensity and ultra-fast protocol are materials recently launched on the market, all studies published to date were carried out *in vitro*. Methodological differences between the different studies may have influenced the results, however, consistency was noticed in the results of the different studies that addressed the same characteristics. As this is a scoping review, it aimed at mapping all research available; bias analysis was not conducted.

## Results

-Study search and selection

**Figure 1:**
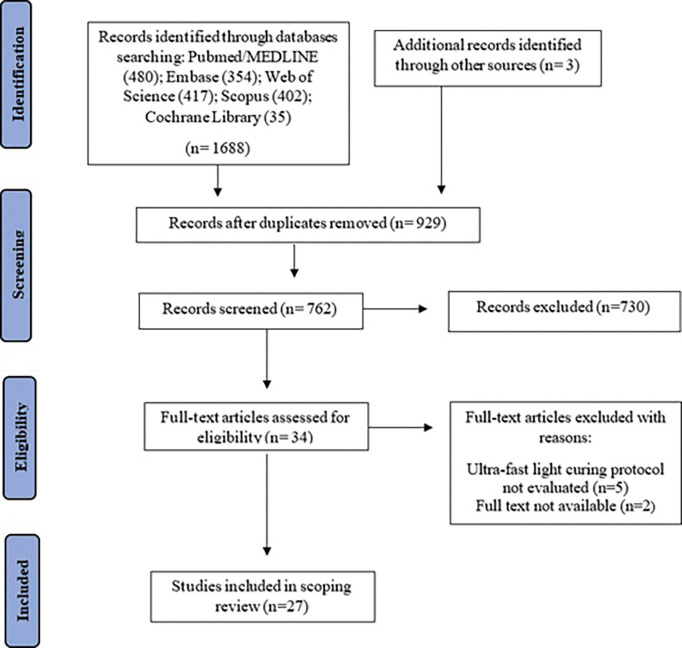
Screening and registration and PRISMA-ScR flow diagram summarizing the article identification and selection processes.

The search of electronic databases resulted in 1.691 articles: PubMed/Medline (480), Embase (354), Web of Science (417), Scopus (402), Cochrane Library (35) and other sources (3). The initial screening removed 929 duplicates; title and abstract reviews excluded 728 studies; 34 studies were selected for full reading. Of these studies, five were excluded because they did not evaluate the behavior of new bulk-fill RBCs with the rapid high-intensity light-curing protocol and two were not full-text available, therefore, 27 studies were eligible for the qualitative synthesis of this scoping review. The agreement between the reviewers in the selection of studies demonstrated “almost perfect agreement” (k=0.871). Figure 1 presents a schematic flowchart summarizing the article selection process.

-Characteristics of the Included Studies

The characteristics and relevant information of the 27 included studies are presented in  Table 2. All the studies included were “*in vitro*”. The articles were published between 2020 and 2023. A total of 2,432 specimens were evaluated. The type of the specimens was dependent on the test performed. Six studies used human extracted teeth (18,30,31,40,42,44); fifteen studies used specimens prepared in molds filled with the composite (14-16,18,19,22,26,32-37,39,41); five studies used disc-shaped specimens (3,21,27,28,29,45) and two studies prepared specimens in resin blocks (3,32). The depth, diameter, and thickness of the samples varied according to evaluation method. The bulk-fill RBCs modified were PowerFill (3,14-16,18,19,21,22,26-29,31) and Powerflow (3,18,19,21,22,26,27,29,30,33).

**Figure 2:**
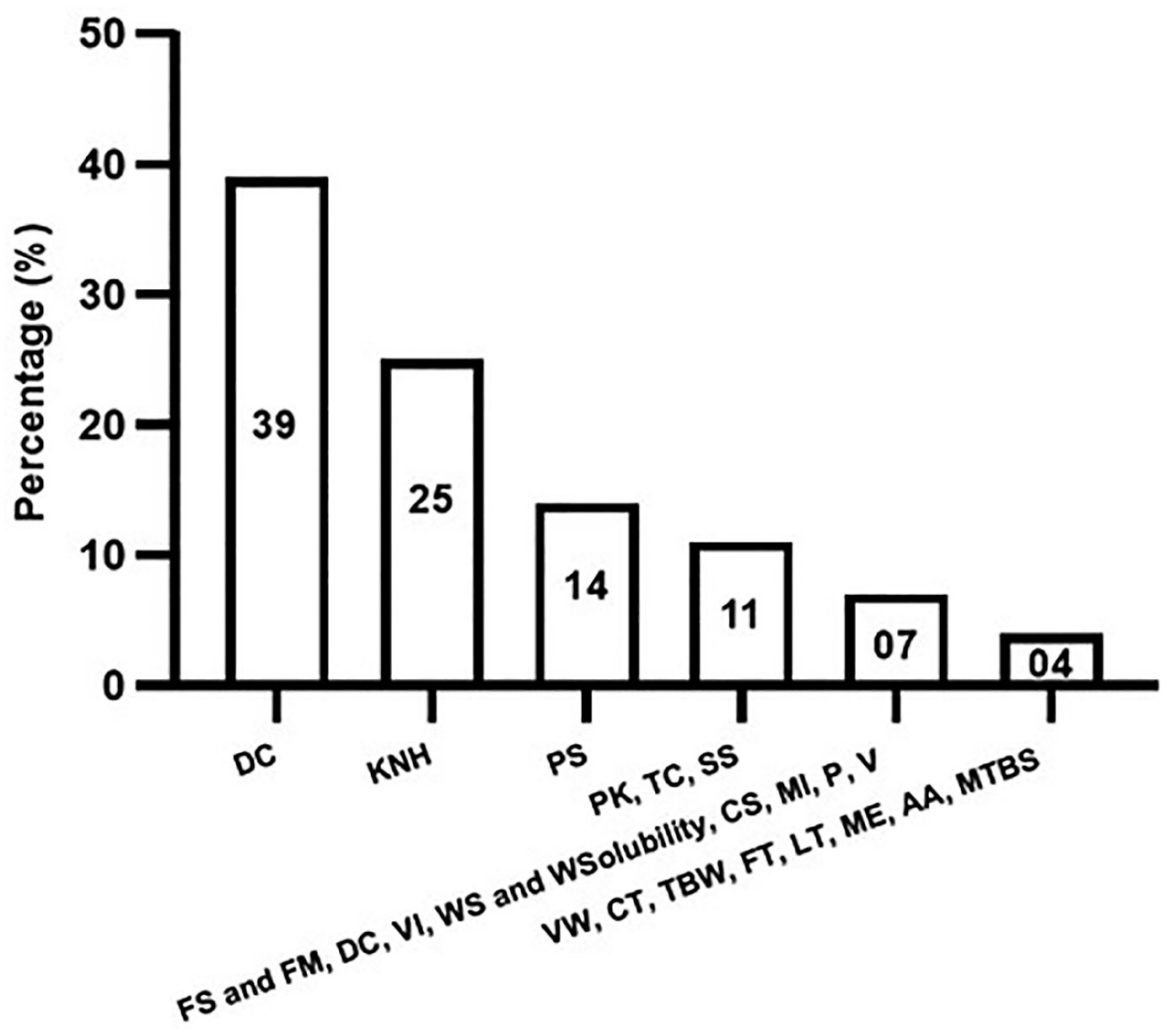
Properties of resin composites evaluated in the experimental *in vitro* studies.

From the included experimental *in vitro* studies, different properties of composite resins were evaluated, including the degree of conversion (3,13,15,18,21,26,32,35-37,39), microhardness (14,15,22,29,32,33,39), polymerization shrinkage (19,21,27,36), polymerization kinetics (18,19,26), temperature changes (15,38,44,45), shrinkage stress (19,21,27), flexural strength and modulus (14,36), depth of cure (14,41), viscoelastic integrity (16,43), water sorption and solubility (32,34), color stability (29,30), marginal integrity (31,40), porosity (3,36), viscosity (16,35), volumetric wear (28), cellular toxicity and viability (16,45), two body wear (32), fracture toughness (32), light transmission (32), monomer elution (36), artificial aging (37), and micro-tensile bond strength (42). Figure 2 summarizes these evaluated properties and the number of studies that addressed them.

-Properties evaluation

The degree of conversion was the property most investigated by the studies, which was adequate when carrying out ultra-fast light curing of the composites of the 3s PowerCure system (26,37), the high viscosity composite showed similar values (14) and was significantly higher than conventional composites (3,15,18,36), two studies reported significantly lower values (21,39). Microhardness was the second-most evaluated property, with lower values for PowerFlow (22) and PowerFill resin composites (14,32,39), although other studies did not demonstrate significant differences (22,15,29,33).

About the polymerization kinetics, significant differences were found for the resin composites of the 3s PowerCure system (14,19). Regarding temperature changes, a greater temperature increase was observed during the light-curing of PowerFill (15) and the new bulk-fill RBCs required the shortest time to reach the maximum recorded temperature (44,45), which was considered accepTable (38) and similar to the temperature observed during conventional light curing (44). Regarding cell viability, the results showed no difference between ultrafast high-intensity polymerization and the standard protocol (45).

The ultra-fast protocol provided adequate viscoelastic characteristics and did not show significant difference to the conventional protocol (16,43), this finding applies to the quasi-static behavior, resistance, and flexural modulus (14). The linear shrinkage and tension generated by the shrinkage of the new bulk-fill RBCs showed behaviors close to those of conventional composites (19,21). The artificial aging of the two composites resulted in inferior mechanical properties (37). Furthermore, these resin composites differed from each other in terms of linear shrinkage, with higher values for PowerFlow (21).

For PowerFill, ultra-fast light curing did not induce cellular toxicity (16), provided moderate viscosity (35), higher light transmission (32), achieved better marginal integrity compared to conventional composites (31), structural integrity improvements (3), comparable microtensile bond strength (42), less polymerization shrinkage (27,36) and tension generated by polymerization shrinkage similar to conventional composites (27). The PowerFlow composite showed an intact internal structure (3), lower values of water sorption and solubility (34) and a depth of cure close to that of conventional light curing (41).

However, ultra-fast light curing impaired water solubility (32), increased porosity (36), color stability was considered clinically unaccepTable (29) and caused greater volumetric wear (28) for the PowerFill. For the PowerFlow resin composite, the tension generated by the polymerization shrinkage was significantly higher (27) and the marginal integrity was significantly lower (40) there was a reduction in the bond strength to dentin (42), higher marginal discoloration (40) and this protocol negatively affected the flexural modulus (37).

-Characteristics of Light Curing Units 

The included studies present the LCU characteristics in  Table 3. The LCU most commonly used in these studies was the Bluephase PowerCure (3,14-16,18-22,26-40,42,43), followed by the Valo Grand Cordless (27,29,38,39,41,45), Elipar S10 (26,32,43,44), SmartLite Pro (38,39,41), Monet (38,39,41), *Pi*nkWave (38,39), Elipar DeepCure LED (15) and modified Bluephase G4 (35). One study used a diode laser (18). LCU properties were evaluated using a radiometer (3,15,26,27,34,36,42-44), spectrophotometer (18,19,21,22,40), spectrometer (14,16,32,37,39,40) and spectroradiometer (35,38). The exposure times for the ultra-fast protocol were 1, 3 or 5s, and those for the conventional protocol were 10 or 20s. For ultra-fast light curing for 1s, the highest irradiance used was 4,645 mW/cm2 (40) 3,770 mW/cm2 in 3s (14) 2,200 mW/cm2 in 5s (38,39). In the conventional protocol, the irradiance ranged from 500 (14,35) to 1,515 mW/cm2 (14). The light emission spectra ranged from 385 (36,38,39,42) to 515nm (36,38,39,42).

## Discussion

Achieving reliable and long-lasting clinical results depends heavily on the properties of restorative materials, particularly their relationship to the aesthetics and function of restorations (33), inadequate or insufficient light-curing can cause problems such as fractures, marginal deterioration, and recurrence of caries (32). To meet the clinician’s needs for performing restorative procedures more quickly and without adverse consequences (22), new bulk-fill RBCs have been developed and formulated specifically for ultra-fast light curing (14). Therefore, this scoping review synthesized scientific evidence, enabling a comprehensive evaluation of the effects of an ultra-fast light-curing protocol on the properties of a new system of bulk-fill RBCs. The composition of the modified bulk-fill RBcs found in this review are in the  Table 4.

It is important to consider that all studies included in this scoping review were laboratory experiments. While these studies can yield precise and reliable results, they cannot fully encompass the clinical challenges that restorative materials face under oral conditions. Therefore, it is essential to develop randomized clinical trials to better understand the effect of ultra-rapid light-curing on new bulk-fill resin composites and its impact on the longevity of dental restorations. The modified bulk-fill RBCs identified in the studies were Tetric PowerFill (low viscosity) and Tetric PowerFlow (high viscosity). The PowerFill uses addition fragmentation chain transfer (AFCT) technology, which generates the ß-allyl sulfone radical after the light-curing process, allowing the reaction to continue, whereas the PowerFlow composite resin does not have differentiated molecules in its composition; its shortened polymerization occurs due to its translucency and the reduced amount of particles (18).

The 3s PowerCure system showed characteristics similar to those of conventional resin composites (14,16,18,21,27,41,43,44), even with ultra-fast light-curing delivering half the energy of the conventional curing, which demonstrates its suitability for clinical application (43). In addition, researchers observed the benefits of using a polywave LCU with high irradiance only for resin composites with alternative photoinitiators (26), which would explain the inferior properties of a conventional resin composite when being light cured with an ultra-fast protocol (32). Conventional bulk-fill resin composites are specifically formulated for moderate intensity light-curing protocols. Consequently, using the ultra-rapid light-curing protocol on these resins prevents them from adequately achieving their intended properties (32).

Depending on the range of composite resins available, and the range of photopolymerization protocols taken into consideration, the choice of restorative material plays a more important role in determining the mechanical properties and degree of conversion than the photopolymerization protocol used (conventional or ultra-fast) (15,22,32,37). In addition, studies that evaluated the mechanical properties such as depth of cure and degree of conversion of new bulk-fill RBCs at different depths have demonstrated that these composites are adequate and presented equivalent results when light-cured either with the ultra-fast protocol or with the conventional protocol (14,29,43). This performance may be related to the presence of benzoyl germanium-based photoinitiators (Ivocerin) as a reinforcement for camphorquinone and the tricyclodecane dimethanol dimethacrylate (DCP) and propoxylated Bisphenol A dimethacrylate particles (43). The bulk-fill RBC Tetric PowerFill showed a degree of conversion equivalent to that of an unmodified bulk-fill RBC regardless of the light-curing mode used, while the Tetric PowerFlow showed superior results with the ISO 4049 protocol (37).

With the aim of adapting these resin composites to ultra-fast light-curing, changes in their compositions were performed (19). The PowerFill uses addition fragmentation chain transfer (AFCT) technology, thus the presence of ß-allyl sulfone polymerization modulator in the organic matrix of this composite allows for the control of its thermal and mechanical properties and is possibly associated with the development of a lower stress generated during polymerization shrinkage (3,27,29), lower loss of fracture toughness (3) and reduced water sorption (34). The ultra-rapid light-curing protocol increased the solubility of the unmodified bulk-fill resin composites (34). The ß-allyl sulfone can enhance the homogeneity of the polymer network, glass transition, and mechanical properties of the new bulk-fill resin composites (27).

The low viscosity composite resin PowerFlow has a lower load content, therefore exhibiting lower strength and durability than PowerFill resin composite (33). This reduced load content also results in greater polymerization shrinkage and a lower modulus of elasticity (27,40). It is possible to speculate that the low bond strength of the PowerFlow resin composite to dentin may be related to the greater polymerization shrinkage and to the ultra-fast protocol, which delivers less energy at greater depths (42), since its translucency is reduced during light-curing because the refractive index of the polymerized matrix changes, and rapidly increases the opacity of the material (18,37). This characteristic also causes a decrease in flexural strength (37). Furthermore, it is believed that shortened light-curing leads to the formation of a larger quantity of residual monomers, resulting in more pronounced discoloration in the new bulk-fill resin composites (29,30). A previous study demonstrated that the bulk-fill RBC Tetric PowerFlow exhibited a higher degree of pigmentation compared to other evaluated bulk-fill RBCs, likely due to differences in the photoinitiators in their compositions (29).

It has been reported that the ultra-fast protocol transiently increases the temperature during light exposure in the dental cavity, possibly causing damage to pulp tissues (38). In the articles that addressed this occurrence, it was observed that the increase in temperature was proportional to the increase in light intensity, which could explain the higher temperature values for the PowerFill and PowerFlow resin composites, although these values were considered clinically acceptable (15,38). Therefore, the application of these 4 mm thick composites in cavities with up to 1 mm of remaining dentin would possibly be clinically safe for use (44). A recently published in-vitro study in simulated pulp chambers with 0.5 mm of remaining dentin and Vero cells concluded that the 3s rapid high-intensity light-curing protocol of bulk-fill RBCs caused a temperature increase greater than 10s and showed cell viability similar to and comparable to the standard protocol (45). Clinical trials are needed to confirm these findings.

The less favorable results regarding the marginal integrity of the PowerFlow resin composite can also be credited to the ultra-fast polymerization speed, which may be responsible for causing very rapid shrinkage, and a short pre-gel phase, preventing deformation during polymerization, and increasing the stresses resulting from high polymerization shrinkage, especially in preparations with high C-factor. To solve the problems related to the significantly lower marginal integrity and microhardness of the flowable resin composite using the ultra-fast protocol, the insertion of a cover layer was suggested, which could attenuate its performance in a clinical environment (40), especially in the region of intense masticatory loads (33). However, the findings regarding marginal integrity should be interpreted with caution, as the type of dental cavity (Class V or Class II) used in the studies may influence the behavior of the modified bulk-fill RBCs (40).

 Laboratory analyses recommended that the clinical tolerance for the use of resin composites of the 3s PowerCure system should be limited to an exposure distance of 5 mm, to avoid insufficient polymerization; when this is not possible, an additional light-curing is advised (14), and cases like this, it was suggested to wait at least the same 3s before the second irradiation to allow thermal recovery and avoid heat propagation (44). It is also of utmost relevance to emphasize that clinicians are responsible for the distance and angle of the LCU tip during the light-curing of the restorative material (44); thus, when using an exposure of 1 or 3s, the operator’s aim must be excellent because if the light is off-target, there will be a significant reduction in energy, resulting in insufficient light-curing with all the consequences arising from a low degree of conversion (39). In addition to this, it is recommended that clinicians use RBCs formulated specifically for rapid light-curing, as they require less energy (32).

The limitations of this review include the level of scientific evidence of the evaluated studies, which included only experimental *in vitro* studies, therefore, the results should be interpreted with caution, as these studies are conducted under controlled conditions and with calibrated operators; the high methodological heterogeneity of the included studies, which made the quantitative analysis of the reported results difficult, and the lack of standardization of specimens and restorative materials used as controls. With this evidence mapping, we suggest future systematic reviews to address more specific inquiries concerning these new resins, such as the impacts of elevated temperatures on dental elements. More studies using natural teeth would be beneficial for performing laboratory analyses that are closer to clinical reality, and, most importantly, clinical trials. Nevertheless, this scoping review allow to better predict the impact of ultra- fast light-curing on the behavior of new bulk-fill RBCs, as can help the design of future clinical studies to provide new information, clinically validate laboratory results, and standardize safe protocols for use.

## Conclusions

The new bulk-fill RBCs can be light-cured with a short exposure time and high intensity, providing a time-saving benefits in clinical practice. This light-curing protocol has shown similar results to standard light-curing in conventional composites. Although, its use should be approached with caution concerning flowable resin composites.

## Figures and Tables

**Table 1:** Search strategy for each database.

Database	Search strategy	Filters
PubMed/ MEDLINE	(composite resins OR composite resin OR resin composite OR resin composites OR resin restoration OR resin restorations OR composite restoration OR composite restorations) AND (bulk fill OR bulk-fill OR bulkfill OR bulk-fill composite OR bulk fill composite OR bulk OR tetric powerfill OR tetric powerflow) AND (led dental curing-light OR led dental curing-lights OR dental curing light OR dental curing lights OR curing light dental OR curing lights dental OR dental curing OR dental curing lights OR high-power led OR high intensity led OR light curing intensity OR fast highintensity light-curing OR rapid high-intensity light-curing OR ultra-fast light-curing OR high irradiance light polymerization OR shortened light-curing)	No filters applied
Embase	('composite'/exp OR composite) AND ('resins'/exp OR resins) OR (('composite'/exp OR composite) AND ('resin'/exp OR resin)) OR (('resin'/exp OR resin) AND ('composite'/exp OR composite)) OR (('resin'/exp OR resin) AND ('composites'/exp OR composites)) OR (('resin'/exp OR resin) AND ('restoration'/exp OR restoration)) OR (('resin'/exp OR resin) AND restorations) OR (('composite'/exp OR composite) AND ('restoration'/exp OR restoration)) OR (('composite'/exp OR composite) AND restorations) AND bulk AND fill OR 'bulk fill' OR bulkfill OR ('bulk fill' AND composite) OR (bulk AND fill AND composite) OR bulk OR (tetric AND powerfill) OR (tetric AND powerflow) AND led AND dental AND 'curing light' OR (led AND dental AND 'curing lights') OR (dental AND curing AND light) OR (curing AND light AND dental) OR (curing AND lights AND dental) OR (dental AND curing) OR (dental AND curing AND lights) OR ('high power' AND led) OR (high AND intensity AND led) OR (light AND curing AND intensity) OR (fast AND 'high intensity' AND 'light curing') OR (rapid AND 'high intensity' AND 'light curing') OR ('ultra fast' AND 'light curing') OR (high AND irradiance AND light AND polymerization) OR (shortened AND 'light curing')	No filters applied
Web of Science	ALL=((composite resins) OR (composite resin) OR (resin composite) OR (resin composites) OR (resin restoration) OR (resin restorations) OR (composite restoration) OR (composite restorations)) AND ALL=((bulk fill) OR (bulk-fill) OR (bulkfill) OR (bulk-fill composite) OR (bulk fill composite) OR (bulk) OR (tetric powerfill) OR (tetric powerflow)) AND ALL=((led dental curing-light) OR (led dental curing-lights) OR (dental curing light) OR (dental curing lights) OR (curing light dental) OR (curing lights dental) OR (dental curing) OR (dental curing lights) OR (high-power led) OR (high intensity led) OR (light curing intensity) OR (fast high-intensity light-curing) OR (rapid high-intensity light-curing) OR (ultra-fast light-curing) OR (high irradiance light polymerization) OR (shortened light-curing))	No filters applied
Scopus	( composite AND resins ) OR ( composite AND resin ) OR ( resin AND composite ) OR ( resin AND composites ) OR ( resin AND restoration ) OR ( resin AND restorations ) OR ( composite AND restoration ) OR ( composite AND restorations ) AND ( bulk AND fill ) OR ( bulk-fill ) OR ( bulkfill ) OR ( bulk-fill AND composite ) OR ( bulk AND fill AND composite ) OR ( bulk ) OR ( tetric AND powerfill ) OR ( tetric AND powerflow ) AND ( led AND dental AND curing-light ) OR ( led AND dental AND curing-lights ) OR ( dental AND curing AND light ) OR ( dental AND curing AND lights ) OR ( curing AND light AND dental ) OR ( curing AND lights AND dental ) OR ( dental AND curing ) OR ( dental AND curing AND lights ) OR ( high-power AND led ) OR ( high AND intensity AND led ) OR ( light AND curing AND intensity ) OR ( fast AND high-intensity AND light-curing ) OR ( rapid AND high-intensity AND light-curing ) OR ( ultra-fast AND light-curing ) OR ( high AND irradiance AND light AND polymerization ) OR ( shortened AND light-curing )	No filters applied
Cochrane Library	(composite resins OR composite resin OR resin composite OR resin composites OR resin restoration OR resin restorations OR composite restoration OR composite restorations) AND (bulk fill OR bulk-fill OR bulkfill OR bulk-fill composite OR bulk fill composite OR bulk OR tetric powerfill OR tetric powerflow) AND (led dental curing-light OR led dental curing-lights OR dental curing light OR dental curing lights OR curing light dental OR curing lights dental OR dental curing OR dental curing lights OR high-power led OR high intensity led OR light curing intensity OR fast highintensity light-curing OR rapid high-intensity light-curing OR ultra-fast light-curing OR high irradiance light polymerization OR shortened light-curing)	No filters applied

**Table 2:** Characteristics of the included studies.

Author, year	Restorative composites tested^a^	n	Study unit	Properties evaluated^b^	Main Results
Algamaiah et al. (2020) (26)	PowerFill, PowerFlow, EvoCeram BF and EvoFlow BF	36	Cylindrical composite samples (4 × 1 or 4 mm)	DC and PK	PowerFill and PowerFlow composites exhibited faster conversion than the controls. PK and the rate of polymerization were influenced by specimen thickness and material viscosity.
Algamaiah et al. (2021) (27)	PowerFill, PowerFlow, EvoCeram BF and EvoFlow BF	40	Disc-shaped composite samples (8 × 1 mm)	PS and SS	PowerFill showed reduced PS and comparable SS to EvoCeram BF. PowerFlow showed significantly higher SS with the 3s protocol.
Alshafi et al. (2023) (28)	Filtek One BF, EvoCeram BF, PowerFill, SonicFill and Filtek Supreme Ultra	50	Disc-shaped composite samples (8 × 1 mm)	VW	PowerFill had a significantly higher wear volume than the other composite resins.
Can et al. (2022) (29)	VisCalor, PowerFill, Fill Up and G-aenial	36	Disc-shaped composite samples (4 × 6 mm)	ΔE00 and VH	All BF composites showed clinically unacceptable ΔE00. There was no difference the bottom VH values of PowerFill.
Erçin et al. (2023) (30)	Omnichroma and PowerFlow	90	Human premolar teeth embedded in plaster models	ΔE00	The most discoloration was observed in coffee solutions for PowerFlow.
Frank et al. (2023) (31)	PowerFill and Prime	40	Class II cavities were prepared in primary human molars	MI	PowerFill achieves similar MI when light-cured with either high-irradiance or regular light-curing modes and achieves better MI than the conventional composite.
Garoushi et al. (2021) (32)	PowerFill and Essencia	122	Mechanical Tests: 3-point bending sticks of composites (2 × 2 × 25 mm); DBC, WSS (1.5 × 4.5 mm); MH (2 and 4 mm); TBD: Composite samples prepared in resin block (20 × 10 × 2 mm); LT: Cylindrical composite samples (1, 2, 3, 4 mm)	FT, WSS, DBC, MH, LT and TDB	The high-intensity light curing protocol (3s) did not endanger the tested properties of PowerFill, except for the surface MH and water solubility. The LT through PowerFill was higher than the conventional composite.
Hayashi et al. (2020) (5)	PowerFill, PowerFlow, EvoCeram BF and EvoFlow BF	32	ID: Cylindrical cavities prepared in composite resin blocks (3 × 4 mm); DC: Disc-shaped composite samples (3 × 5 mm)	ID and DC	PowerFlow showed an intact internal structure and great DC ratio on through the depth, despite a slightly lower DC at the top of the specimen. PowerFill has improved its structural integrity and DC.
Ilie,Watts. (2020) (12)	PowerFill and EvoCeram BF	32	DC: Cylindrical composite samples (3 × 2 or 4 mm); FS, FM: (2 × 2 × 18 mm); VH, DOC: (6 × 10 mm)	FS, FM, DC, VH and DOC	The PowerFill was shown to induce comparable properties to those of a conventional BF composite. Differences were evidenced only in faster initial PK in the RAFT system. Small differences in VH and FM were related.
Ilie, Diegelmann. (2021) (14)	PowerFill	24	Cylindrical composite samples (4 × 5 mm)	CT, QS and SVI	The 3s polymerization protocol induced no CT and an equivalent QS and viscoelastic mechanical behavior as with conventional curing protocols in PowerFill.
Jakupovic et al. (2023) (33)	Evetric, Prime, EvoFlow, PowerFill and PowerFlow	40	Cylindrical composite samples; (6 mm × 2 or 4 mm)	MH	The MH of RBCs is more dependent on material composition than on light-curing protocol.
Klaric et al. (2021) (34)	Filtek One BF, PowerFill, PowerFlow, SDR Plus BF Flow and Z250	30	Cylindrical composite samples (9 × 2 mm)	WSS	The 3s polymerization protocol increased the solubility of all materials. PowerFlow exhibited the lowest WSS values compared to other materials.
Labrie et al. (2022) (35)	PowerFill, Aura BF and Heliomolar	108	Cylindrical composite samples (0.2, 2 and 4 mm thick)	DC and VISC	PowerFill followed exposure reciprocity between irradiance levels of 500 to 3000 mW/cm^2^, and has a moderate viscosity.
Lempel et al. (2023) [36]	PowerFill and Filtek One BF	30	Cylindrical composite samples (6 × 4 mm)	DC, ME, PS and PO	High-irradiance rapid 3-s curing of PowerFill resulted in inferior results for PS and PO compared to a conventional composite.
Marovic et al. (2021) (37)	PowerFill, PowerFlow, Filtek One BF and SDR Plus BF Flow	40	FS and FM: Cylindrical composite samples (16 × 2 × 2 mm); DC: (2 or 4 mm)	FS, FM, DC and AA	Mechanical properties deteriorated by increasing depth and aging. 3s polymerization protocol was sufficient for PowerFill, however, it negatively affected FM of materials, whereas its influence on FS and DC was material dependent.
Maucoski et al. (2023) (38)	Filtek One BF, Filtek BF Flow, PowerFill and PowerFlow	360	Non-retentive Class I and V cavities prepared in human molar	TA	Short 1 to 3s exposures produced acceptable temperature rises, regardless of the composite.
Maucoski et al. (2023) (39)	Filtek One BF, PowerFill, Filtek BF Flow and PowerFlow	576	Cylindrical composite samples DC: (4 × 4 mm); VH: (2 × 2 mm)	DC and VH	The shorter exposure times delivered lower DC and VH values. PowerFill and PowerFlow was less affected than conventional composites when they received a high irradiance for a short time.
Negovetic et al. (2023) (40)	Filtek One BF, PowerFill and PowerFlow	24	Cylindrical composite samples (3 × 4 mm)	PK and DC	DC values of sculptable composites were significantly higher to the flowable composites.
Par et al. (2020) (21)	EvoFlow BF, PowerFlow, X-tra Base, PowerFill, Filtek One BF, EvoCeram BF and Ceram.X	56	Disc-shaped composite samples (1.5 mm thick)	LS, SF, and DC	The flowable composites showed significantly higher LS and SF compared to sculptable composites. The ultra-fast light curing composites showed shrinkage parameters comparable to other composites.
Par et al. (2020) (20)	EvoFlow BF, PowerFlow, X-tra Base, PowerFill, Filtek One BF, EvoCeram BF and Ceram.X	56	Cylindrical composite samples (6 × 2 or 4 mm)	MH	Flowable composites showed high-intensity curing diminished MH and improving crosslinking density. In sculptable composites, the MH was unaffected, crosslinking density was reduced.
Par et al. (2021) (40)	PowerFlow, X-tra Base, PowerFill and Filtek One BF	80	Class V cavities prepared on buccal surfaces of human molars	MI	The flowable composites light-curing using the high-intensity protocol showed a significantly inferior MI, it may compromise the tooth-restoration interface.
Par et al. (2022) (17)	SureFil, Filtek One BF, PowerFill, PowerFlow, Filtek Supreme Flow and EvoFlow Mosaic, Herculite, Filtek	108	Cylindrical composite samples (6 × 1.5 mm)	PK, LS and SS	PowerFill and PowerFlow differed from the other investigated materials with regard to PK, while showing comparable behavior in terms of LS and SS.
Rocha et al. (2022) (41)	Supreme Ultra, EvoCeram, Admira F, Estelite Q, Mosaic- EN, SDR flow+, PowerFlow and X-tra Fil	150	Cylindrical composite samples (6 × 10 mm)	DOC	PowerFlow reached a similar DOC (values above the 4 mm threshold), regardless of the irradiation protocol used.
Steffen et al. (2022) (42)	PowerFill, PowerFlow, Filtek One BF and SDR Flow +	64	Extracted human molars were ground to dentin	μTBS	The use of 3 s polymerization protocol has no negative influence on the μTBS of the investigated high-viscosity BF composites. However, it may reduce the μTBS of PowerFlow.
Watts, Algamaiah. (2020) (43)	PowerFill and PowerFlow, EvoCeram BF and EvoFlow BF	48	Cylindrical composite samples (4 × 4 mm)	SVI	PowerFill and PowerFlow demonstrated an acceptable level of surface integrity and viscoelastic characteristics, evidencing their suitability for clinical application.
Wang et al. (2021) (13)	Filtek BF, PowerFill, Beautiful BF and Admira Fusion-Xtra	100	Cylindrical composite samples (5 × 4, 3, 2 or 1 mm)	TA, MH and DC	The high-intensity protocol caused the highest peak temperature irrespective of the composite types. The highest peak temperature was from PowerFill. There was no significant difference in MH. PowerFill showed the highest DC.
Yang et al. (2021) (44)	Filtek BF, PowerFill, Beautiful BF and Admira Fusion-Xtra	60	Prepared cavities in the first human molar at 4 measurement locations: (0, 2, and 4 mm from the top and 1 mm into dentin)	TA	Regardless of light-curing protocols, PowerFill and PowerFlow took the shortest time to reach the maximum temperature. The ultra-fast protocol showed generally comparable temperature rise to the standard protocol.
Miranda et al. (2024) (45)	Filtek BF Flow and Tetric PowerFlow	40	Dentin discs from human molars, included in artificial pulp chambers	TA, CV	The 3s rapid high-intensity light-curing protocol of bulk-fill composite resins caused a temperature increase greater than 10s and showed cell viability similar and comparable to the standard protocol.

**Table 3:** Light Curing Units characteristics.

Author, year	LCU^a^	LCU valuation method	Irradiance and irradiation time	Emission spectrum
Algamaiah et al. (2020) (24)	)PowerCure and Elipar S10	Radiometer	PowerCure (3 and 5s): 3000 and 2000 mW/cm^2^ S10 (20s): 1000 mW/cm^2^	–
Algamaiah et al. (2021) (25)	PowerCure and Valo	Radiometer	PowerCure (3 and 10s): 3000 and 1200 mW/cm^2^ Valo (3s): 3200 mW/cm^2^	–
Alshafi et al. (2023) (26)	PowerCure	–	3s: 3050 mW/cm^2^	–
Can et al. (2022) (27)	Valo	–	3 and 10–20s: 3200 and 1000 mW/cm^2^	–
Erçin et al. (2023) (28)	PowerCure	–	3s: 3000 mW/cm^2^	–
Frank et al. (2023) (29)	PowerCure	–	3 and 10s: 3000 and 1200 mW/cm^2^	–
Garoushi et al. (2021) (30)	PowerCure e and Elipar S10	Spectrometer	PowerCure (3s): 2400 mW/cm^2^ S10 (20s): 1600 mW/cm2	PowerCure: 400–500 nm S10: 430–480 nm
Hayashi et al. (2020) (5)	PowerCure	Radiometer	3 and 10s: 3000 and 1000 mW/cm^2^	410–460 nm
Ilie,Watts. (2020) (12)	PowerCure	Spectrometer	3s: 3770 mW/cm^2^ (0 mm), 2693 mW/cm^2^ (5 mm) and 1176 mW/cm^2^ (10 mm) 10s: 1515 mW/cm^2^ (0 mm), 1083 mW/cm^2^ (5 mm), and 500 mW/cm^2^	412–455 nm
Ilie, Diegelmann. (2021) (14)	PowerCure	Spectrometer	3s: 3720 mW/cm2 10–20s: 1446 mW/cm	410–452 nm
Jakupovic et al. (2023) (31)	PowerCure	–	3 and 10s: 3440 and 1340 mW/cm^2^	390–500nm
Klaric et al. (2021) (32)	PowerCure	Radiometer	3 and 20s: 3053 and 1193 mW/cm^2^	–
Labrie et al. (2022) (33)	PowerCure and Bluephase G4	Spectroradiometer	Pcure (3s): 3140 mW/cm^2^ G4 (12 and 24s): 3000, 2000 and 1000 mW/cm^2^	PCure: 408–448 nm G4: 449 nm
Lempel et al. (2023) (34)	PowerCure	Radiometer	3s: 3150 mW/cm2 5s: 1950 mW/cm2 10 and 20s: 1180 mW/cm2/cm^2^	385–515 nm
Marovic et al. (2021) (35)	PowerCure	Spectrometer	3 and 20s: 2700 and 950 mW/cm^2^	404–447 nm
Maucoski et al. (2023) (36)	Monet, PinkWave, SL Pro, Valo and PowerCure	Spectroradiometer	Monet (1-3s): 2000–2400 mW/cm^2^ PinkWave (3 and 20s): 1515 and 1720 mW/cm^2^ SL Pro (20s): 1200 mW/cm^2^ Valo (5 and 20s): 2200 and 1100 mW/cm2 PowerCure (3 and 20s): 3050 and 1200 mW/cm^2^	Monet: 450 nm PinkWave: 395–900 nm SL Pro: 450–480 nm Valo: 385–515 nm PowerCure: 385–515 nm
Maucoski et al. (2023) (37)	Monet, PinkWave, SL Pro, Valo and PowerCure	Spectrometer	Monet (1–3s): 2000–2400 mW/cm2 PinkWave (3 and 20s): 1515 and 1720 mW/cm^2^ SL Pro (20s): 1200 mW/cm^2^ Valo (5 and 20s): 2200 and 1100 mW/cm^2 ^PowerCure (3 and 20s): 3050 and 1200 mW/cm^2^	Monet: 450 nm PinkWave: 395–900 nm SL Pro: 450–480 nm Valo: 385–515 nm PowerCure: 385–515 nm
Negovetic et al. (2023) (16)	PowerCure and diode laser	Spectroradiometer	3s, 5s and 10s: 3000, 2000 and 1000 mW/cm^2^	449 nm
Par et al. (2020) (19)	PowerCure	Spectroradiometer	3 and 10s: 3440 and 1340 mW/cm^2^	390–500 nm
Par et al. (2020) (20)	PowerCure	Spectroradiometer	3 and 10s: 3440 and 1340 mW/cm^2^	390–500 nm
Par et al. (2021) (38)	PowerCure	Spectroradiometer	3 and 10s: 3440 and 1340 mW/cm^2^	390–500 nm
Par et al. (2022) (17)	PowerCure	Spectroradiometer	3, 5 and 10s: 2850, 2050 and 1100 mW/cm^2^	390–500 nm
Rocha et al. (2022) (39)	Monet, SL Pro and Valo	Spectrometer	Monet (1 and 3s): 4645 mW/cm^2^ SL Pro (10 and 20s): 1182 mW/cm^2^ Valo (10 and 20s): 1299 mW/cm^2^	SL Pro: 462 nm Valo: 462 nm Monet: 452 nm
Steffen et al. (2022) (40)	PowerCure	Radiometer	3 and 10s: 2850 and 1160 mW/cm^2^	385–515 nm
Watts, Algamaiah. (2020) (41)	PowerCure and Elipar S10	Radiometer	PowerCure (3 and 5s): 3000 and 2000 mW/cm^2^ S10 (20s): 1200 mW/cm^2^	–
Wang et al. (2021) (13)	PowerCure and Elipar	Radiometer	PowerCure (3 and 5s): 3050 and 2100 mW/cm^2^; (10 and 20s): 1200 mW/cm^2^ Elipar (20s): 1470 mW/cm^2^	–
Yang et al. (2021) (42)	PowerCure and Elipar S10	Radiometer	PowerCure (3s): 3000 mW/cm^2^ PowerCure and S10 (10s): 1200 mW/cm^2^	–
Miranda et al. (2024) (45)	Valo Grand	Spectrometer	Valo Grand (3s): 3200 mW/cm^2^ Valo Grand (10s): 1000 mW/cm^2^	-

aBrand names of light curing units cited: PCure, Bluephase PowerCure (Ivoclar Vivadent); Elipar S10 (3M/ESPE); SL Pro, SmartLite Pro (Dentisply Sirona); Valo, Valo Grand Cordless (Ultrradent); Monet (AMD Lasers); Bluephase G4, Modified Bluephase G4 (Ivoclar Vivadent); Elipar, Elipar DeepCure LED (3M/ESPE); PinkWave (Vista Dental Products).

**Table 4:** New bulk-fill RBCs compositions.

New Bulk-fill RBCs	Composition
Tetric PowerFlow (Ivoclar Vivadent)	Bis-GMA, Bis-EMA and UDMA, plus aromatic dimethacrylate and tricyclodocane dimethanol dimethacrylate. The monomer matrix comprises a higher percentage (approx. 34%) of the composite. camphorquinone/amine and Ivocerin initiator. Barium aluminium silicate glass, an Isofiller, ytterbium fluoride and a spherical mixed oxide amounting to an overall filler content of approximately 71%. Aessencio Technology: increase opacity that the composite undergoes during polymerization.
Tetric PowerFill (Ivoclar Vivadent)	Bis-GMA, Bis-EMA and UDMA, plus aromatic dimethacrylate and tricyclodocane dimethanol dimethacrylate. The monomer matrix comprises a low percentage (approx. 18%) of the composite. camphorquinone/amine and Ivocerin initiator. : barium aluminium silicate glass, an Isofiller, ytterbium fluoride and a spherical mixed oxide, amounting to an overall filler content of approximately 79%. The composition of Tetric PowerFill was further optimized by including a (β-allyl sulfone) addition fragmentation chain transfer (AFCT) reagent.

Bis-GMA: Bisphenol A glycidyl methacrylate; BIS-EMA: Bisphenol A Ethoxylate DimethacrylateUDMA: Urethane Dimethacrylate;

## Data Availability

The datasets used and/or analyzed during the current study are available from the corresponding author.
